# Cumulative Live Birth Rates Following Stimulation With Corifollitropin Alfa Compared With hp-hMG in a GnRH Antagonist Protocol in Poor Ovarian Responders

**DOI:** 10.3389/fendo.2019.00175

**Published:** 2019-03-22

**Authors:** Joaquín Errázuriz, Alessia Romito, Panagiotis Drakopoulos, Billie Frederix, Analissa Racca, Neelke De Munck, Herman Tournaye, Michel De Vos, Christophe Blockeel

**Affiliations:** ^1^Centre for Reproductive Medicine, Universitair Ziekenhuis Brussel, Vrije Universiteit Brussel, Brussels, Belgium; ^2^Departamento de Ginecología y Obstetricia, Clínica Alemana, Facultad de Medicina, Universidad del Desarrollo, Santiago, Chile; ^3^Department of Ginecological-Obstetrical and Urological Sciences, Sapienza University, Rome, Italy; ^4^Department of Surgical and Clinical Science, Faculty of Medicine and Pharmacy, Vrije Universiteit Brussel, Brussels, Belgium; ^5^Academic Unit of Obstetrics and Gynecology, Azienda Ospedaliera Universitaria San Martino (IRCCS), University of Genoa, Genova, Italy; ^6^Department of Obstetrics and Gynecology, University of Zagreb-School of Medicine, Zagreb, Croatia

**Keywords:** poor responders, corifollitropin alfa, hp-hMG, Bologna criteria, cumulative live birth rate

## Abstract

**Background:** Bologna criteria poor ovarian responders have a very low prognosis. Although, it has been proposed that LH supplementation could be beneficial in women with previous hypo-response to FSH. There are no studies comparing the cumulative live birth rates (LBRs) between corifollitropin alfa (CFA) and highly purified human menopausal gonadotrophin (hp-hMG).

**Objective:** To compare cumulative LBRs in Bologna poor ovarian responders undergoing ovarian stimulation with CFA followed by hp-hMG vs. hp-hMG alone in a GnRH antagonist protocol.

**Design:** This is a retrospective cohort study. We included in total 917 poor responders fulfilling the Bologna criteria for poor ovarian response (POR) at a university-affiliated tertiary center from January 2011 until March 2017. Patients were administered either fixed daily doses of 300–450 IU of hp-hMG (group A) or a single dose of 150 μg of CFA followed by daily injections of ≥300 IU of hp-hMG from Day 8 of stimulation until the day of ovulation trigger (group B), in a fixed GnRH antagonist protocol.

**Results:** LBRs after fresh embryo transfer (ET) were similar in group A 71/510 (14%) and B 42/407 (10%). Cumulative LBR per cycle was significantly higher in group A (16.9%) compared to group B (11.8%); (*P* = 0.03). However, logistic regression analysis showed no association between the type of gonadotropin administered and cumulative LBR. Only age was significantly associated with cumulative LBR (OR = 0.93, *P* = 0.007).

**Conclusion:** Cumulative LBRs are similar in Bologna poor responders stimulated with CFA followed by hp-hMG compared to hp-hMG monotreatment in an antagonist protocol.

## Introduction

Poor ovarian response (POR) is observed in at least 10% of infertile women but the incidence rises with advancing age. Reproductive treatment in these patients remains a major challenge in fertility research (1), mainly because of the low live birth rate of approximately 6% per cycle in this population, irrespective of the treatment protocol used (2).

An important hurdle for improving outcomes in poor responders is the historical lack of consensus with regard to the diagnosis of POR, with studies using a variety of definitions, which could hamper the clinical value of inter-study comparison and meta-analysis in this group of patients (3). The introduction of the Bologna criteria in 2011 represents a major step toward proper identification of this group of women and the adoption of these criteria paved the way for studies using an unanimous and formal definition (4).

Different treatment protocols for the management of Bologna poor responders have been evaluated so far, but currently no single stimulation protocol stands out as unequivocally effective (5). Corifollitropin alfa (CFA) is a long acting gonadotropin that has been designed as a sustained follicle stimulant with the ability to simplify ovarian stimulation, given that a single subcutaneous injection can replace the first seven injections of any follicle-stimulating hormone (FSH) preparation (6). This is particularly important if we consider the psychological distress and the high dropout rates observed in IVF patients (7). Moreover, it has been demonstrated that in normal responders, CFA results in enhanced follicular recruitment and an increased oocyte yield (8), with pregnancy rates comparable to those of recombinant FSH (rFSH) stimulation (9).

However, despite these beneficial results in normal responders, very few studies, mainly pilot studies, have evaluated the role of CFA followed by rFSH or hp-hMG in poor responders, with some of them showing promising results (2, 10). A major limitation of these studies is that they included a small number of individuals, without a comparison group.

Given the paucity of evidence and the urgent need to improve the reproductive outcome of this specific group of infertile women, the aim of our study was to analyse cumulative LBR in Bologna poor responders treated with CFA followed by hp-hMG compared with hp-hMG monotreatment in a GnRH antagonist protocol.

## Materials and Methods

### Study Design

This was a retrospective single-center cohort study, aiming to evaluate cumulative LBR in Bologna poor responders treated either with CFA followed by hp-hMG or hp-hMG alone, in a GnRH antagonist protocol. Demographic and clinical data of intracytoplasmic sperm injection (ICSI) cycles were collected in women attending the Centre for Reproductive Medicine, Universitair Ziekenhuis Brussel, Belgium, from 1st January 2011 until 1st March 2017 (Ethical Committee of Brussels University Hospital approval B.U.N 1432001836906).

### Eligibility Criteria

Data were retrieved from patients who fulfilled the Bologna criteria (4). More specifically, at least two of the following criteria had to be present: (i) advanced maternal age (≥40 years); (ii) a previous POR (≤3 oocytes with a conventional stimulation protocol); (iii) an abnormal ovarian reserve test (i.e., AFC < 7 follicles or AMH < 1.1 ng/ml). Anti-Müllerian hormone (AMH) was measured in a previous menstrual cycle, irrespective of the cycle day. Antral follicle count (AFC) was measured on day 2–4 of a previous menstrual cycle. Patients were allocated to the two stimulation protocols based on the physician's discretion.

Further inclusion criteria were: age between 18 and 43 years old, body mass index (BMI) of 17 to 35 kg/m^2^, presence of both ovaries, absence of any untreated endocrine abnormality and no use of oral contraceptives nor estrogen priming prior to ovarian stimulation. Patients who underwent pre-implantation genetic testing (PGT), conventional *in vitro* fertilization (IVF) cycles for fertility preservation and natural or modified natural IVF cycles were excluded from the analysis. In order to avoid crossovers between treatments, each patient contributed with only one cycle to the dataset. Finally, women who had remaining cryopreserved embryos from their stimulation cycle and who had not delivered a live birth at the moment of the data collection were excluded from this analysis.

### Treatment Protocol

#### Ovarian Stimulation

On Day 2 of the menstrual cycle patients were administered either a single subcutaneous dose of 150 μg CFA (Elonva®; MSD, Oss, The Netherlands) or started a course of seven fixed daily doses of 300 IU up to 450 IU of hp-hMG (Menopur®; Ferring, Saint-Prex, Switzerland). In the CFA/hp-hMG group, daily doses of ≥300 IU of hp-hMG were administered from Day 8 of stimulation until the day of ovulation triggering, when required. Hp-hMG dose was adjusted according to the stimulation response that was monitored with serial measurements of serum estradiol and transvaginal ultrasonic evaluation of follicle number and size.

Pituitary down-regulation was performed with daily administration of GnRH-antagonist (ganirelix; Orgalutran®; MSD, Oss, The Netherlands) starting on Day 6 of stimulation.

#### Ovulation Trigger and Luteal Phase Support

Final oocyte maturation was triggered with either highly purified urinary or recombinant human chorionic gonadotrophin (hCG), (Pregnyl®, MSD, Oss, The Netherlands; or Ovitrelle®; Merck Serono Europe Ltd, London, UK) when at least two follicles reached 17 mm in mean diameter. In case of monofollicular development, patients were allowed to proceed to oocyte retrieval.

Cumulus-oocyte complexes (COC) were collected by transvaginal aspiration 36 h after the hCG administration followed by insemination via the ICSI procedure as described previously (11).

Luteal phase support consisted of vaginal progesterone (Utrogestan®; Besins Healthcare, Paris, France), administered daily (three times 200 mg per day) and initiated on the day after oocyte retrieval and continued for at least 7 weeks in case of a positive pregnancy test.

### Embryo Transfer

Ultrasound-guided fresh embryo transfer (ET) was performed 3 or 5 days after oocyte retrieval with a maximum of 3 embryos transferred. When at least 4 embryos of top quality (at least 7 cells with maximum 10% fragmentation) or good quality (at least 6 cells with maximum 20% fragmentation) were present on Day 3, embryo culture was extended until Day 5, followed by fresh ET on Day 5. Blastocyst quality was categorized as excellent (AA), good (AB, BA, BB), fair (BC, CB), or poor (CC) based on trophectoderm and inner cell mass quality scores. Only good quality embryos were cryopreserved (12). Otherwise, ET took place on Day 3.

### Cryopreservation

On Day 3 or Day 5, supernumerary good quality embryos (or all embryos in case of a freeze all approach) were vitrified using closed high security vitrification straws (Cryo Bio System®, Paris, France) combined with dimethylsulphoxide and ethylene glycol bis (succinimidyl succinate) as cryoprotectants (Irvine Scientific® Freeze Kit, Canada) (12). Good-quality Day 3 embryos were defined as embryos that reached the 6-cell stage with <20% fragmentation. Good-quality Day 5 embryos were defined as having trophectoderm and inner cell mass quality scores of at least AB, BA, or BB.

### Frozen–Thawed Embryo Transfer

Frozen ET, following warming of vitrified embryos, was performed either in a natural cycle, with or without hCG triggering, or in an artificial cycle. The decision regarding the type of preparation for the frozen ET cycle was made by the physician, based on the menstrual cycle pattern of the patient. The number of embryos transferred (one or two) in the frozen-thawed cycles complied with Belgian regulatory guidelines and patients' individual preference (13).

### Primary Outcome

The primary outcome was the cumulative LBR defined as the delivery of a liveborn (>22 weeks of gestation) in the fresh or in the subsequent frozen-thawed cycles (14). Only the first delivery was considered in the analysis. Patients underwent follow-up until exhaustion of all embryos derived from the stimulation cycle.

### Secondary Outcomes

The secondary outcomes included biochemical pregnancy (a detection of beta hCG in serum), clinical pregnancy (a pregnancy diagnosed by ultrasonographic visualization of one or more gestational sacs, including the ectopic pregnancies), ongoing pregnancy (diagnosed by ultrasonographic visualization of an intrauterine sac with embryonic pole demonstrating cardiac activity at 10 weeks of gestation), and live birth (delivery of a liveborn after 22 weeks of gestation, following the fresh ICSI cycle only).

### Statistical Analysis

Continuous data are presented as mean ± standard deviation (SD) or as median and interquartile range, as appropriate. Categorical data are described by number of cases and percentages. Continuous variables were compared with the use of independent *t*-test or Mann-Whitney U-test, depending on the normality of the distribution, and categorical variables were compared with the chi-square or Fisher's exact test, as appropriate. The level of significance was set at *P* < 0.05.

To identify characteristics that may be associated with the cumulative LBR, multivariate logistic regression analysis was performed with the cumulative live birth as the dependent variable and type of treatment as the main independent variable (hp-hMG alone or CFA followed by hp-hMG). Other candidate variables were age, BMI, number of oocytes retrieved, day of transfer (Day 3 vs. Day 5) and number of embryos transferred in the fresh cycle. All variables were simultaneously entered into the logistic regression model. The likelihood of cumulative LBR after ICSI is presented as an odds ratio (OR) with standard error (SE) and 95% confidence interval (CI). All statistical tests used a two-tailed α of 0.05. All analyses were performed using SPSS version 24.0.

## Results

### Baseline Characteristics of the Study Population

In total, data from 917 patients fulfilling the Bologna criteria were analyzed and divided into two groups: patients in group A received hp-hMG (*n* = 510) and patients in group B received CFA followed by hp-hMG (*n* = 407). Patients' baseline characteristics between the two groups were similar regarding age, BMI and AFC (Table 1). However, AMH and basal FSH were significantly different between both groups (0.7 ng/ml vs. 0.5 ng/ml; *P* < 0.001 and 8.9 IU/l vs. 10 IU/l; *P* < 0.001, respectively). The number of previous attempts was similar in both groups (1 vs. 1; *P* = 0.218). The Baseline characteristics are presented in Table 1.

**Table 1:** Baseline characteristics.

	**Hp-hMG group A (*n* = 510)**	**CFA + hp-hMG group B (*n* = 407)**	***P-*value**
Age (years)	39 (36–41)	39 (36–41)	0.837
BMI (kg/m^2^)	24 (22–28)	24 (22–28)	0.667
Number of previous attempts	1 (0–3)	1 (0–3)	0.218
AFC	5 (3–7)	5 (3–7)	0.272
AMH (ng/ml)	0.7 (0.3–1.0)	0.5 (0.2–0.9)	<0.001
Serum FSH (IU/L)	8.9 (7.0–11.4)	10.0 (7.6–13.0)	<0.001

*Data are expressed as median (IQR). BMI, body mass index; AFC, antral follicle count; AMH, anti-müllerian hormone; FSH, follicle stimulating hormone*.

### Ovarian Stimulation Characteristics

The cycle characteristics are presented in Table 2. The duration of stimulation was significantly different between group A and B (9.3 vs. 10.1 days, respectively; *P* < 0.001).

**Table 2:** Characteristics of the ovarian stimulation.

	**Hp-hMG group A (*n* = 510)**	**CFA + hp-hMG group B (*n* = 407)**	***P-*value**
Total days of stimulation	9 (8–11)	10 (9–11)	<0.001
COCs	4 (2–6)	3 (2–5)	<0.001
MII	3 (2–5)	3 (1–4)	0.004
Fertilization rates (%)	75 (50–100)	80 (57–100)	0.114
Cycle with ET, *n* (%)	386 (76%)	279 (69%)	0.016
Number of embryos transferred	2 (1–2)	2 (1–2)	0.401
**DAY OF TRANSFER**, ***N*** **(%)**			
Day 3	349 (90%)	244 (88%)	0.339
Day 5	37 (10%)	35 (12%)	
At least one top quality embryo among patients with ET Day 3, *n* (%)	595 (28.7%)	444 (31.4%)	0.088
At least one top quality embryo among patients with ET Day 5, *n* (%)	32 (1.5%)	24 (1.7%)	0.724
Patients with cryopreserved embryos, *n* (%)	128 (25%)	88 (22%)	0.2
Number of embryos cryopreserved	2 (1–3)	1 (1–2)	0.007

Data are expressed as median (IQR) or as number (percentage). COCs, cumulus-oocyte complexes; MII, metaphase II oocytes. Fertilization rates: (number of MII fertilized/MII^*^100).

The number of COCs retrieved was significantly higher in group A compared to group B (4.6 vs. 3.7; *P* < 0.001). Conjointly, significant differences were observed in the number of metaphase II (MII) oocytes (3.6 vs. 3.0; *P* = 0.004). Fertilization rates were comparable between groups.

Characteristics of ET and embryo development are presented in Table 2. Overall, 252 (27%) patients did not have an ET either because there were no oocytes retrieved or due to a lack of good quality embryos to transfer. 76% of patients in the CFA/hp-hMG group and 69% of patients in the hp-hMG group had an ET (*P* = 0.016), with a similar number of embryos transferred in both groups. Most ETs (90%) took place on Day 3. The percentage of patients with cryopreserved embryos was comparable between the groups [128 patients (25%) after hp-hMG and 88 patients (22%) after CFA/hp-hMG, *P* = 0.2]. A significant difference was noted in the number of embryos cryopreserved between the two treatments [2 (1-3) for hp-hMG and 1 (1-2) for CFA/hp-hMG, *P* = 0.007].

### Reproductive Outcomes

Reproductive outcomes are presented in Table 3. Biochemical pregnancy [131/510 (26%) vs. 74/407 (18%); *P* = 0.007], clinical pregnancy [120/510 (23%) vs. 66/407 (16%); *P* = 0.006] and ongoing pregnancy rates [88/510 (17%) vs. 45/407 (11%); *P* = 0.008) were significantly higher in the hp-hMG treatment group compared to the CFA/hp-hMG group. However, live birth rates were not statistically different between hp-hMG [71/510 (14%)] and CFA/hp-hMG treatment group [42/407 (10%); *P* = 0.09]. The cumulative LBR after fresh ET and subsequent frozen ETs from the same stimulation cycle was significantly higher following hp-hMG stimulation [86/510 (16.9%) vs. 48/407 (11.8%); *P* = 0.03).

**Table 3:** Reproductive outcomes.

	**Hp-hMG group A (*n* = 510)**	**CFA+ hp-hMG group B (*n* = 407)**	***P-*value**
Biochemical pregnancy, *n* (%)	131 (26%)	74 (18%)	0.007
Clinical pregnancy, *n* (%)	120 (23%)	66 (16%)	0.006
Ongoing pregnancy, *n* (%)	88 (17%)	45 (11%)	0.008
Live birth, *n* (%)	71 (14%)	42 (10%)	0.1
CLBR, *n* (%)	86 (16.9%)	48 (11.8%)	0.03

*Data are expressed as number (percentage). CLBR, cumulative live birth rates*.

### Multivariate Regression Analysis of Cumulative Live Birth Rates

Table 4 presents the variables associated with cumulative LBR and their estimated ORs, SE, and CI. Multivariable logistic regression analysis showed that age was the only parameter associated with cumulative LBR [OR = 0.934 (CI 95% 0.889–0.982; *P* = 0.007)]. The type of treatment (hp-hMG monotreatment or CFA followed by hp-hMG) was not significantly associated with cumulative LBR.

**Table 4:** Multivariate logistic regression with odds ratios for cumulative live birth.

**Cumulative live birth**	**Odds ratio**	**95% confidence interval**	***P*-value**	**SE**
Age	0.934	0.889–0.982	0.007	0.025
BMI	1.013	0.970–1.058	0.558	0.022
COCs	1.059	0.982–1.143	0.137	0.039
**TREATMENT TYPE**				
hp-hMG	1	–	–	
CFA/hp-hMG	0.809	0.537–1.217	0.309	0.209
**DAY OF FRESH ET**				
Day 3	1	–	–	0.307
Day 5	1.325	0.727–2.417	0.358	
Number of embryos transferred in the fresh cycle	1.065	0.797	1.422	0.148

*BMI, body mass index; COCs, cumulus-oocyte complexes; ET, embryos transfer; SE, standard error*.

### Multivariate Regression Analysis of Fresh Live Birth Rates

Supplementary Table I presents the variables associated with fresh LBR and their ORs (SE) and CI.

Age was the only variable associated with fresh LBR [OR = 0.907 (CI 95% 0.862–0.955; *P* < 0.001)].

## Discussion

This study encompasses one of the largest series of Bologna poor responders and is the first to evaluate cumulative LBRs after ovarian stimulation using a single injection of CFA followed by hp-hMG vs. daily administration of hp-hMG, in poor ovarian responders fulfilling the Bologna criteria. Based on our findings, fresh LBR and cumulative LBR were comparable between groups after adjustment for relevant confounders.

Live birth rates in our study are lower than those in a small previous pilot study by our group suggesting that CFA followed by hp-hMG in a long GnRH agonist protocol appears to be a promising treatment option for Bologna poor ovarian responders (2). In the aforementioned study, LBRs were 17% in the 47 poor responders allocated to the long agonist protocol, while our findings suggest a lower LBR of 10%. However, it should be mentioned that there was no comparison group in the previous pilot study, the sample size was very small and cumulative LBRs were not evaluated. These limitations are inherent to a pilot study and may explain the fact that a recent RCT comparing ongoing pregnancy rates in young Bologna poor responders stimulated with CFA/hp-hMG vs. FSH in an antagonist protocol (15), failed to replicate the promising findings of an earlier pilot study (10). Pilot studies are linked to selection and confounding biases which may substantially affect the validity of the results (16) and for this reason it was clearly underscored in the previous pilot studies that these data were preliminary and in need of further validation.

CFA is a novel recombinant fertility hormone with prolonged follicle-stimulating activity used for ovarian stimulation in ART. CFA is composed of FSH fused with the C-terminal peptide of the beta-subunit of hCG and exhibits a slower absorption and a longer elimination half-life in comparison to rFSH (17). All studies conducted in poor responders comparing CFA with other gonadotropins used a dosage of 150 μg of CFA (10, 15, 18). This dose is not based on clinical data, but is extrapolated from evidence derived from normal responders (19, 20). Data from randomized controlled trials and retrospective studies suggest that this dosage (150 μg of CFA) may be promising in poor responders (10, 18). The hypothesis of including hp-hMG in the comparison between groups came from the observation that better clinical outcomes may be obtained when hCG is added (21, 22). However, although several meta-analyses have demonstrated a benefit of LH addition in ovarian stimulation protocols (23, 24), the effect, as also the timing of hp-hMG addition to FSH in POR remains controversial (25, 26). Furthermore, it is known that LH and hCG have different molecular features leading to hormone-specific intracellular signaling cascades (27). A recent meta-analysis showed that this is linked to a different clinical action of LH and hCG in ovarian stimulation (28). Therefore, LH activity is not the same as hCG action and further evidence is warranted.

Studies published so far on CFA treatment in patients with low ovarian reserve have been characterized by a limited number of patients. Several of them reported that stimulation with CFA may result in a slightly higher number of oocytes in comparison with other gonadotropins, probably due to higher serum levels of FSH that are reached during the first days of the stimulation (10, 15). In addition, there is evidence that CFA supplemented with ≥300 IU/day of rFSH or hp-hMG from the 8th day of stimulation may be a promising alternative (10, 18, 19, 29). Nonetheless, we could not confirm these findings, given that although we detected significant differences in the cumulative LBR in the unadjusted analysis, logistic regression analysis showed no association between the type of gonadotropin administrated and cumulative LBR.

A major strength of our study lies in its large sample size and in the choice of cumulative live birth rate as primary outcome parameter, which is a highly relevant clinical outcome parameter for patients (30). Furthermore, we included poor responders according to the Bologna criteria, and this should be emphasized given the clinician's reluctance to use the Bologna criteria in studies of poor responders (31). Nevertheless, our study has a number of limitations. The retrospective study design should be kept in mind when interpreting results and prevents us from drawing firm conclusions. Although multivariate analysis was performed to adjust for all known confounders, it cannot be excluded that non-apparent sources of bias might still be present. Patients were allocated to groups based on clinicians' discretion, so selection bias may still have occurred. Furthermore, it should be stated that although our study included Bologna poor responders, differences in baseline characteristics could have impacted the prognosis of our patients. Nonetheless, Bologna criteria are not presumed to predict clinical prognosis, but rather define a group of patients with low ovarian reserve (32). For instance, AMH levels were higher in group A than in group B and this difference (albeit not clinically relevant) may have modulated the secondary endpoints. However, in the multivariate regression analysis, we adjusted for the number of oocytes retrieved (which correlates with AMH levels), therefore this imbalance was taken into account for our final outcome: cumulative LBR. Finally, differences in the secondary endpoints of our study could also be the result of polymorphisms in gonadotropins' receptors/genes, which are known to be linked to ovarian response (33–36).

In conclusion, ovarian stimulation in an antagonist protocol using CFA followed by hp-hMG did not appear to result in superior clinical outcomes compared to hp-hMG monotreatment, in terms of fresh and cumulative LBR, in Bologna criteria poor ovarian responders. Prospective randomized controlled trials are required to validate these results in order to have an impact on clinical practice.

## Data Availability

All datasets generated for this study are included in the manuscript and/or the Supplementary Files.

## Author Contributions

JE, PD, and BF are responsible for the concept design and wrote the manuscript. JE, BF, ND, AlR, and PD performed the data extraction and statistical analysis. AnR, ND, HT, MD, and CB contributed to the interpretation of the results and editing of the manuscript.

### Conflict of Interest Statement

The authors declare that the research was conducted in the absence of any commercial or financial relationships that could be construed as a potential conflict of interest.
