# Supplementary material for: Cumulative Live Birth Rates Following Stimulation With Corifollitropin Alfa Compared With hp-hMG in a GnRH Antagonist Protocol in Poor Ovarian Responders
Source: Front Endocrinol (Lausanne). 2019 Mar 22;10:175. doi: 10.3389/fendo.2019.00175 (PMC6439614; doi:10.3389/fendo.2019.00175)
Supplement: Supplementary file 1 [file Table_1.docx]

**Supplementary table I Multivariate logistic regression with odds ratios for fresh live birth rate**

| **Cumulative live birth** | **Odds ratio** | **95% confidence interval** | ***P*-value** | **SE** |
| --- | --- | --- | --- | --- |
| Age | 0.907 | 0.862-0.955 | <0.001 | 0.026 |
| BMI | 1.008 | 0.963-1.055 | 0.726 | 0.023 |
| COCs | 0.967 | 0.888-1.054 | 0.448 | 0.044 |
| Treatment  hp hMG  CFA/ hp hMG | 1  0.783 | -  0.509 - 1.203 | -  0.265 | 0.219 |
| Day of fresh ET  Day 3  Day 5 | 1  1.606 | -  0.857-3.012 | -  0.140 | 0.321 |
| Number of embryos transferred in the fresh cycle | 1.436 | 1.063-1.940 | 0.018 | 0.153 |
